# Impact of Ramadan Fasting on the Severity of Symptoms Among a Cohort of Patients With Gastroesophageal Reflux Disease (GERD)

**DOI:** 10.7759/cureus.36831

**Published:** 2023-03-28

**Authors:** Abdullah H Bohamad, Walaa A Aladhab, Sawsan S Alhashem, Mohammed S Alajmi, Turki Alhumam, Danah J Alqattan, Ahmed M Elshebiny

**Affiliations:** 1 Internal Medicine, King Faisal University, Al Ahsa, SAU; 2 Internal Medicine and Endocrinology, King Faisal University, Al Ahsa, SAU

**Keywords:** gastro-esophageal disease, gerd pathophysiology, gastrointestinal symptoms, gastro-oesophageal reflux, prevalence of gerd, clinical variation, pain severity, ramadan fasting, fasting ramadan, gastroesophageal reflux disease (gerd)

## Abstract

Introduction: Gastroesophageal reflux disease (GERD) is a condition caused by the reflux of stomach contents into the esophagus. Heartburn, chest discomfort, and regurgitation are the main symptoms. Medications, surgical procedures, and lifestyle modification are considered treatment options. Fasting is believed to be one of the lifestyle modifications that helps minimize GERD symptoms. Muslims abstain from eating, drinking, and smoking from dawn until dusk. The objectives of our study were to investigate the relationship between fasting and GERD symptoms and evaluate how fasting affects GERD symptoms in Saudi Arabia.

Methodology: This was a longitudinal study that selected GERD patients for its consecutive sampling. The patients answered the questionnaires at two separate times: once during Ramadan and once after Ramadan. A validated gastroesophageal reflux disease health-related quality of life (GERD-HRQL) self-administered survey was used.

Result: After Ramadan, heartburn symptoms significantly decreased, particularly when lying down. Overall, the 45-point heartburn score decreased from 17.9 during Ramadan to 14.3 thereafter. The regurgitation score decreased from 12.3 during Ramadan to 9.9 after fasting, with statistical significance (P = .049). Although satisfaction was much higher after Ramadan (17% vs. 15.1%), there was no statistical significance (P = .422), and 45.3% of the patients were satisfied with their health state during Ramadan compared to 34% after Ramadan. There was no relationship between the severity of GERD symptoms before or after fasting and the type of food, the timing of eating, or the amount of food consumed.

Conclusion: The results suggested that Ramadan fasting may improve GERD symptoms. However, more studies are required to validate these results and comprehend the underlying mechanisms.

## Introduction

Gastroesophageal reflux disease (GERD) is a disease resulting from the reflux of gastric contents into the esophagus or beyond [1]. The main symptoms of GERD are heartburn and regurgitation. However, GERD can be accompanied by a variety of other symptoms, including chest pain, dysphagia, coughing, hoarseness, throat clearing, burning, and wheezing [2]. GERD is often diagnosed clinically based on the presence of symptoms and the responses to medication [3]. GERD is a widespread condition that is most prevalent in North America. The prevalence of GERD in North America ranged from 18.1% to 27.8% [2]. The prevalence of GERD in Saudi Arabia is 28.7% [4]. Currently, several risk factors have been identified, including esophageal dysmotility, hiatal hernia, age greater than 50 years, obesity, low socioeconomic status, tobacco use, excess alcohol consumption, pregnancy, and postprandial supination [5-9]. Treatment options include lifestyle modifications, medical management, and surgical therapy [10-15]. Fasting is one of the lifestyle changes that can help reduce GERD symptoms. Muslims during Ramadhan abstain from eating, drinking, and smoking from dawn until sunset [16]. Muslims who abstain from smoking during Ramadan may reduce GERD symptoms. A study showed that an intermittent fasting regimen of 16 hours daily for four days could improve the symptoms of both regurgitation and heartburn [17]. Another study showed that intermittent fasting can improve the symptoms of both regurgitation and heartburn [18]. A study conducted showed that GERD symptoms are milder in participants during Ramadhan than during non-Ramadhan months [17]. Another study showed no effect on GERD symptoms [19]. Only two studies were done in Indonesia and Iran about the effect of Ramadan fasting on GERD symptoms. Our study aims to fill the gap in literature reviews to expand the knowledge of the correlation between fasting and symptoms of GERD and assess the effect of fasting on GERD symptoms in Saudi Arabia.

## Materials and methods

Study design

This longitudinal study was conducted based on consecutive sampling and choosing GERD patients who had been clinically diagnosed by a gastroenterologist. The questionnaire was administered to study participants on two different occasions, namely, the first time in the last week of Ramadan and the second time in the first week after two consecutive months of Ramadan.

Study population and instruments

Patients over the age of 18 years who had been clinically diagnosed with GERD by a gastroenterologist and were willing to participate fully in two rounds of the questionnaire met the inclusion criteria. Exclusion criteria were patients younger than 18 years who had not been clinically diagnosed with GERD by a gastroenterologist and were not willing to engage fully in two rounds of questioning. The online survey was used to gather information on GERD symptoms using the validated version of the self-administered gastroesophageal reflux disease health-related quality of life (GERD-HRQL). The survey was conducted from June 2022 to August 2022, receiving 51 responses. The survey was translated into Arabic since it is the main language of Saudi Arabia. Four minutes were needed to complete the survey. The survey was divided into four sections. The first section began with participant consent. In the second section, participants were asked five questions about demographic data. In the third section, participants were asked about personal habits, such as smoking and heartburn medication use, the amount and type of food they eat, and the interval between meals and sleep. The final section contains the GERD-HRQL which has 15 items that addressed heartburn, regurgitation, swallowing difficulties, and the impacts of GERD on daily living. A five-point Likert scale was used to rate the items (no symptoms = 0, incapacitating symptom = 5). Additionally, the survey included a question to evaluate the patients' satisfaction on three scales: satisfied, neutral, and dissatisfied (score range: 1-3). The total number of questions is 15, with six questions for heartburn and six questions for regurgitation; the final score ranges are 0-75, 0-30, and 0-30, respectively.

Ethical consideration

The King Faisal University Committee on biomedical ethics approved this study; the approved ethical code was KFU-REC-2022-APR-EA000558. Participants gave their informed consent to participate in the study. The goals of the study were known to the participants. The confidentiality agreement was maintained, and no personal data were collected. 

Data analysis

The data were collected, reviewed, and then fed to Statistical Package for Social Sciences, version 21 (SPSS, IBM Corp., Armonk, NY). All statistical methods were two-tailed with an alpha level of 0.05, and a P-value less than or equal to 0.05 was considered significant. The mean scores for different GERD-related domains (heartburn and regurgitation) were calculated, and we used the mean scores to determine the overall score for each and the question's score (out of five points). Descriptive analysis was done by prescribing frequency distribution and percentages for study variables including participants' biodemographic data, medical history, and medications. Also, the effect of fasting on GERD-related symptoms was assessed for heartburn and regurgitation using a paired t-test. The effect of food type, amount of food, and timing of food intake during and after fasting on the severity of GERD symptoms was assessed using a one-way analysis of variance (ANOVA). Cross-tabulation for detecting dietary habits during and after fasting among GERD patients was done using the McNemar test.

## Results

A total of 53 GERD patients were included. The ages of the patients ranged from 16 to 67 years, with a mean of 33.7 ± 15.2 years. Exactly 31 (58.5%) patients were female, and 48 (90.5%) were Saudi. Participants' education levels were as follows: 33 (62.3%) had university-level education or above, 18 (34%) had secondary-level education, and two had a lower level of education. A total of eight (15.1%) had chronic health problems, 10 (18.9%) were asthmatic, and seven (13.2%) were smokers. A total of 26 (49.1%) used antacids, and 24 (45.3%) used other medications (Table 1).

**Table 1:** Biodemographic data of study patients with GERD GERD: Gastroesophageal reflux disease.

Biodemographic data	No	%
Age in years		
<25	23	43.4%
25-39	13	24.5%
40+	17	32.1%
Gender		
Male	22	41.5%
Female	31	58.5%
Nationality		
Saudi	48	90.6%
Non-Saudi	5	9.4%
Education		
Below secondary	2	3.8%
Secondary/diploma	18	34.0%
University or above	33	62.3%
Have chronic health problems		
Yes	8	15.1%
No	45	84.9%
Have asthma		
Yes	10	18.9%
No	43	81.1%
Smoking		
Yes	7	13.2%
No	46	86.8%
Use antacids medications		
Yes	26	49.1%
No	27	50.9%
Use other medications		
Yes	24	45.3%
No	29	54.7%

GERD health-related quality of life among patients during and after fasting

Table 2 shows the effects of Ramadan fasting on clinical symptoms in patients with GERD. In terms of heartburn when lying down, the score was significantly lower after Ramadan (2.3 to 1.87 out of 5; P= .043). Also, when patients stood up, they noticed a significant decrease in heartburn (1.68 to 1.25 out of 5; P= .049), patients noticed a significant decrease after meals (2.49 to 1.83 out of 5; P = .006), and patients noticed a significant decreased interrupting sleep (2.19 to 1.28 out of 5; P = .002). The result showed that the heartburn score was reduced from 17.9 during fasting to 14.3 after fasting, with statistical significance (P= .027). Considering regurgitation, the regurgitation score decreased from 2.28 to 1.66 out of 5 (P= .008), and regurgitation woke patients from sleep (1.85 to 1.34; P= .048). The regurgitation score was reduced from 12.3 before fasting to 9.9 after fasting, with statistical significance (P= .049) (Table 2).

**Table 2:** GERD-HRQL among patients during and after fasting *P < 0.05 (significant). P: Paired t-test; GERD: Gastroesophageal reflux disease; GERD-HRQL: Gastroesophageal reflux disease health-related quality of life.

GERD-HRQL	During fasting		After fasting		P-values
	Mean	SD	Mean	SD	
Heartburn					
How bad is the heartburn?	2.13	1.16	1.79	1.20	.101
Heartburn when lying down?	2.30	1.20	1.87	1.33	.043*
Heartburn when standing up?	1.68	1.22	1.25	1.00	.049*
Heartburn after meals?	2.49	1.32	1.83	1.20	.006*
Does heartburn change your diet?	2.23	1.64	1.91	1.61	.224
Does heartburn wake you from sleep?	2.19	1.51	1.28	1.18	.002*
Do you have difficulty swallowing?	1.26	1.16	.96	1.33	.188
Do you have a gassy or bloating feeling?	2.47	1.40	2.19	1.48	.328
If you take redux medication, does this affect your daily life?	1.13	1.30	1.21	1.49	.802
Overall score	17.9	7.8	14.3	8.4	.027*
Regurgitation					
How bad is the regurgitation?	2.28	1.32	1.66	1.34	.008*
Regurgitation when lying down?	2.23	1.17	1.81	1.40	.113
Regurgitation when standing up?	1.60	1.21	1.26	1.27	.177
Regurgitation after meals?	2.25	1.24	2.04	1.45	.434
Does regurgitation change your diet?	2.06	1.49	1.75	1.64	.257
Does regurgitation wake you from sleep?	1.85	1.28	1.34	1.36	.048*
Overall score	12.3	5.6	9.9	7.3	.049*

Dietary habits during and after Ramadan among GERD patients

A total of 73.6% of the GERD patients had an intermediate amount of food during fasting compared to 66% after Ramadan, while 15.1% had a lot of food (up to satiety) during Ramadan versus 7.5% after Ramadan (P= .048). As for the interval between the last meal of the day and bedtime, there was no significant difference during and after Ramadan, and for food types on the patients' tables during and after Ramadan, the most frequent were starches (77.4% and 75.5%, respectively) and protein (67.9% and 73.6%, respectively; P= .409 (Table 3).

**Table 3:** Dietary habits during and after fasting among GERD patients *P < 0.05 (significant). P: McNemar test; GERD: Gastroesophageal reflux disease.

Dietary habits	During Ramadan		After Ramadan		P-values
	No	%	No	%	
How much food do you eat during meals?					.048*
Little	6	11.3%	14	26.4%	
Intermediate	39	73.6%	35	66.0%	
A lot (up to satiety)	8	15.1%	4	7.5%	
How long is the interval between the last meal of the day and bedtime?					.752
Half an hour	3	5.7%	2	3.8%	
1 hour	12	22.6%	13	24.5%	
2 hours	18	34.0%	16	30.2%	
3 hours or more	20	37.7%	22	41.5%	
What are the most common foods on your table?					.409
Vegetables	24	45.3%	23	43.4%	
Starches	41	77.4%	40	75.5%	
Fired food	26	49.1%	20	37.7%	
Protein	36	67.9%	39	73.6%	
Fruits	21	39.6%	25	47.2%	
Dairy products	16	30.2%	17	32.1%	
Sweets	17	32.1%	22	41.5%	

Dietary habits during fasting and their relationship with the severity of GERD symptoms

There was no significant association between the type of food, food intake time, and amount of food and the symptoms of GERD, with the higher heartburn score being for those who ate little food (19.2, 7.0), those who had their meals one hour before bedtime (19.6, 7.5), and those who had dairy products (20.5, 7.5). When it comes to regurgitation, those who ate a lot of food (13.9, 6.9 out of 10), those who ate three hours before bedtime (13.3, 5.1), and those who ate dairy products (14.6, 4.4) all had higher scores (Table 4).

**Table 4:** Dietary habits during fasting and their relationship with the severity of GERD symptoms GERD: Gastroesophageal reflux disease.

Dietary habits during fasting	Heartburn score			Regurgitation score		
	Mean	SD		Mean	SD	
How much food do you eat during meals in Ramadan?			.898			.421
Little	19.2	7.0		12.8	5.1	
Intermediate	17.6	7.8		11.8	5.5	
A lot (up to satiety)	18.3	9.2		13.9	6.9	
How long is the interval between the last meal of the day and bedtime during Ramadan?			.664			.428
Half an hour	15.3	8.1		10.0	6.0	
1 hour	19.6	7.5		13.1	6.9	
2 hours	16.4	8.5		11.0	5.2	
3 hours or more	18.6	7.7		13.3	5.1	
What are the most common foods on your table during Ramadan?			.527			.865
Vegetables	18.4	8.4		13.3	6.1	
Starches	18.1	8.3		12.6	5.7	
Fired food	17.2	8.3		12.5	5.8	
Protein	17.4	7.6		12.8	5.5	
Fruits	18.1	8.2		13.2	5.8	
Dairy products	20.5	7.5		14.6	4.4	
Sweets	18.8	8.7		12.0	7.4	

Dietary habits after fasting and their relationship with the severity of GERD symptoms

There was no significant association between the type of food, food intake time, and amount of food and the symptoms of GERD severity, with the higher heartburn score being for those who ate little food (15.4, 11.1), those who have food two hours before bedtime (15.9, 5.8), and those who had sweets (15.7, 7.0). When it comes to regurgitation, those who ate little food (12.7, 10) have a higher score than those who ate two hours before bedtime (10.5, 3.5) and those who ate sweets (10.5, 6.8) (Table 5).

**Table 5:** Dietary habits after fasting and their relationship with the severity of GERD symptoms GERD: Gastroesophageal reflux disease.

Dietary habits after fasting	Heartburn score			Regurgitation score		
	Mean	SD		Mean	SD	
How much food do you eat during meals in Ramadan?			.059			.187
Little	15.4	11.1		12.7	10.0	
Intermediate	14.3	7.3		9.1	5.8	
A lot (up to satiety)	10.5	7.3		6.5	5.8	
How long is the interval between the last meal of the day and bedtime during Ramadan?			.827			.975
Half an hour	12.0	9.9		10.0	12.7	
1 hour	13.4	7.5		9.9	6.5	
2 hours	15.9	5.8		10.5	3.5	
3 hours or more	13.9	10.5		9.4	9.5	
What are the most common foods on your table during Ramadan?			.776			.607
Vegetables	12.5	8.4		9.0	8.6	
Starches	13.9	8.5		9.6	7.6	
Fired food	14.5	9.1		10.1	7.0	
Protein	13.2	7.7		8.9	7.3	
Fruits	13.6	7.7		10.2	7.9	
Dairy products	12.5	6.5		7.8	5.5	
Sweets	15.7	7.0		10.5	6.8	

## Discussion

Summary of the result

Heartburn improved significantly after Ramadan, especially heartburn that woke participants from sleep (P = 0.002) and after meals (P = 0.006). This study indicates that there was no strong connection between food intake time and the type and severity of GERD symptoms, both during fasting and non-fasting months. The study's results indicate no strong connection between the food intake time and the type and severity of GERD symptoms. High scores of heartburn and regurgitation were found in participants who ate little food, had food close to bedtime, and consumed dairy products or sweets; these associations were not statistically significant.

Discussion

After Ramadan, heartburn improved significantly, particularly heartburn that woke participants from sleep (P = 0.002) and after meals (P = 0.006). This result was unexpected as the literature review found that fasting improves GERD symptoms [17], but this could be due to the changes in eating patterns. In Ramadan, people eat heavy and fatty meals for Iftar (a meal eaten after sunset) after fasting for 14-15 hours, which could explain the aggravation of heartburn after meals. Other authors' publications have also demonstrated the link between hefty meals and heartburn [20-22]. This observation is consistent with the biomechanics theory, which states that excessive stomach wall contraction, such as that caused by large meals and air building up in the fundus of the stomach when eating, weakens the mechanisms governing the lower esophageal sphincter's activities (LES) [23]. Additionally, most Muslims go to sleep less than two hours after Suhoor (a meal eaten before sunrise), which may explain the significant decrease in heartburn waking participants from sleep after Ramadan. Regurgitation improved significantly after Ramadan. This contrasts with a study by Mardhiyah et al., in which GERD symptoms were less severe during Ramadan than they were throughout other months [17]. The results of this study indicate that there was no strong connection between the food intake time and the type and severity of GERD symptoms, both during fasting and non-fasting months. Although higher scores of heartburn and regurgitation were found in participants who ate little food, had food close to bedtime, and consumed dairy products or sweets, these associations were not statistically significant. This finding is consistent with several previous studies that investigated the relationship between food intake time and the type and severity of GERD symptoms.

A review found that there is limited evidence to support dietary factors and GERD symptoms [24]. However, it is important to note that the relationship between dietary factors and GERD symptoms is complex and may be influenced by individual differences such as body weight, age, and lifestyle habits. Further research is needed to fully understand the potential effects of food intake time and type of GERD symptoms. There was no significant association between the amount of food intake and the severity of GERD symptoms during both fasting and non-fasting months. This finding is consistent with previous studies that have investigated the relationship between food intake and GERD symptoms. Similarly, a study shows that there was no significant association between meal amount and GERD symptoms [25]. However, other studies have reported conflicting results. For example, a study found that larger meal sizes were associated with increased GERD symptoms, and other reports reported that late-night meals and large meals were associated with increased GERD symptoms [26,21]. The finding that there was no significant association between the type of food and the severity of GERD symptoms during both the fasting and non-fasting months is inconsistent with several other studies. This shows there are some types of food that are associated with GERD symptoms, such as fatty, fried, sour, and spicy food or products; orange and grapefruit juice; tomatoes and tomato preserves; chocolate; coffee or tea; carbonated beverages; and alcohol as triggers for GERD [27]. Also, the finding that heartburn and regurgitation scores were higher among those who had little food, those who had food close to bedtime, and those who had dairy products are somewhat inconsistent with some other studies. Overall, the relationship between meal timing, type of food, and GERD symptoms appears to be complex and may vary depending on individual factors. This study's results align with some, but not all, previous studies on the relationship between food intake and GERD symptoms. Further research is needed to clarify the relationship between these variables and determine the optimal dietary modifications for reducing GERD symptoms.

Limitations

Our study limitations include a small size sample size. The questionnaire was distributed online rather than through in-person interviews, which might have affected the participants' answers. The answers to some questions are not objective; rather, they depend on the individual's experience, for example, the question about how much food they consume.

Recommendation

We recommend that a larger study be conducted with a more varied and representative sample size to maximize the generalizability of the results. In-person interviews should be used to gather more specific information and limit the misunderstanding of some questions, which could misinterpret the results. Lastly, to improve the questionnaire of this study, researchers may investigate adopting objective techniques to collect data on food intake, such as food diaries or dietary recalls to increase the accuracy of the data.

## Conclusions

This study investigated the effects of Ramadan fasting on clinical symptoms in patients with GERD. Results showed that GERD symptoms such as heartburn and regurgitation were significantly reduced during and after Ramadan, but no significant association was found between GERD symptoms and dietary habits such as type of food, food intake time, or amount of food. The study found that 45.3% of GERD patients were satisfied with their health status during Ramadan, while 34% were satisfied after Ramadan, with no significant difference. The results suggest that Ramadan fasting may have a positive impact on GERD symptoms, but further research is needed to confirm these findings and understand the underlying mechanisms.
